# Genomic and transcriptomic analysis of the thermophilic lignocellulose-degrading fungus *Thielavia terrestris* LPH172

**DOI:** 10.1186/s13068-021-01975-1

**Published:** 2021-06-03

**Authors:** Monika Tõlgo, Silvia Hüttner, Peter Rugbjerg, Nguyen Thanh Thuy, Vu Nguyen Thanh, Johan Larsbrink, Lisbeth Olsson

**Affiliations:** 1 Wallenberg Wood Science Centre, Department of Biology and Biological Engineering, Chalmers University of Technology, SE-412 96 Gothenburg, Sweden; 2 Division of Industrial Biotechnology, Chalmers University of Technology, SE-412 96 Gothenburg, Sweden; 3 Center for Industrial Microbiology, Food Industries Research Institute, Thanh Xuan, Hanoi, Vietnam

**Keywords:** Biomass degradation, Carbohydrate active enzymes, Cellulose, Filamentous fungi, LPMO, Thermostable enzymes, Transcriptome, Xylan

## Abstract

**Background:**

Biomass-degrading enzymes with improved activity and stability can increase substrate saccharification and make biorefineries economically feasible. Filamentous fungi are a rich source of carbohydrate-active enzymes (CAZymes) for biomass degradation. The newly isolated LPH172 strain of the thermophilic Ascomycete *Thielavia terrestris* has been shown to possess high xylanase and cellulase activities and tolerate low pH and high temperatures. Here, we aimed to illuminate the lignocellulose-degrading machinery and novel carbohydrate-active enzymes in LPH172 in detail.

**Results:**

We sequenced and analyzed the 36.6-Mb genome and transcriptome of LPH172 during growth on glucose, cellulose, rice straw, and beechwood xylan. 10,128 predicted genes were found in total, which included 411 CAZy domains. Compared to other fungi, auxiliary activity (AA) domains were particularly enriched. A higher GC content was found in coding sequences compared to the overall genome, as well as a high GC3 content, which is hypothesized to contribute to thermophilicity. Primarily auxiliary activity (AA) family 9 lytic polysaccharide monooxygenase (LPMO) and glycoside hydrolase (GH) family 7 glucanase encoding genes were upregulated when LPH172 was cultivated on cellulosic substrates. Conventional hemicellulose encoding genes (GH10, GH11 and various CEs), as well as AA9 LPMOs, were upregulated when LPH172 was cultivated on xylan. The observed co-expression and co-upregulation of genes encoding AA9 LPMOs, other AA CAZymes, and (hemi)cellulases point to a complex and nuanced degradation strategy.

**Conclusions:**

Our analysis of the genome and transcriptome of *T. terrestris* LPH172 elucidates the enzyme arsenal that the fungus uses to degrade lignocellulosic substrates. The study provides the basis for future characterization of potential new enzymes for industrial biomass saccharification.

**Supplementary Information:**

The online version contains supplementary material available at 10.1186/s13068-021-01975-1.

## Background

The biorefinery concept represents the basis for a more sustainable bio-based economy aimed at converting abundant renewable biomass sources into energy and value-added products. Today, around 40 lignocellulosic biorefineries operate across Europe [1]. Even though lignocellulose is a potential biomass resource, its degradation is impeded by high lignin content and heterogeneity of its polysaccharide constituents [2, 3]. Biomass saccharification into fermentable monomeric sugars by enzymatic hydrolysis is a crucial step in a biorefinery, but it is hindered by the high cost of enzymes. Indeed, enzymes have been estimated to add 1 USD/gallon to the cost of bioethanol produced from poplar. Thus, there is strong demand for improved enzyme activity and stability [4].

Various potential industrial enzymes exist in nature [5] and the Kingdom Fungi, with more than a million species, represents a particularly rich source [6]. As major biomass degraders, fungi possess a broad array of enzymes suitable for lignocellulose degradation, which are often secreted in large quantities [7]. Thermophilic and thermo-tolerant fungi are especially interesting, as their enzymes can usually endure harsh conditions used in the industry, such as extreme temperatures or pH and harsh solvents [8, 9]. For example, biomass hydrolysis by the industrial *Trichoderma reesei* enzymes in a separate hydrolysis–fermentation process (SHF), is performed at 45–50 °C and pH 5 and therefore additional enzymes that are added to this process to enhance hydrolysis further should show high activity under the same conditions. Thermostable enzymes can lower industrial processing costs as they can achieve faster reaction rates, greater stability, and are more easily adjustable to various setups [10]. Yet, it should be kept in mind that novel enzymes from thermophiles are not necessarily thermostable when produced heterologously [11].

*Thielavia terrestris* (*syn Thermothielavioides terrestris* [12]) is a well-known filamentous fungus identified in 1983 as a potential source of thermostable industrial enzymes based on successful (hemi)cellulase assays [13, 14]. The species is a thermophilic saprobic Ascomycete isolated mainly from soil and compost in Asia [15–17] and from a cave cricket species in North America [18]. *T. terrestris* also played a pivotal role in the discovery of the cellulase-boosting effect of the glycoside hydrolase family 61 (GH61) proteins [19–23], today known as auxiliary activity family 9 (AA9) lytic polysaccharide monooxygenases (LPMOs) [24]. As described by Merino and Cherry [19], cultivation broth from *T. terrestris* primed for cellulase production showed striking synergy in degrading pre-treated corn stover when supplemented with the enzyme cocktail Celluclast. In 2011, *T. terrestris* strain NRRL 8126 and *Myceliophthora thermophila* ATCC 42464 were the first thermophiles whose genomes were fully sequenced and the first filamentous fungi with known telomere-to-telomere genome sequences [25]. The same study showed that *T. terrestris* could potentially degrade all plant cell wall polysaccharides and the fungus hydrolyzed alfalfa straw at temperature optima of 40 °C and 60 °C. As shown by proteomics analyses [26] and detailed biochemical characterization [15, 16, 20, 27–33], *T. terrestris* produces an array of biomass-degrading enzymes. However, no study has elucidated the gene expression mechanisms underlying the lignocellulolytic machinery of the fungus in detail.

In recent years, it has become clear that genetic or gene expression differences between fungal strains of the same species are not uncommon [17, 34–37]. In this study, we set out to sequence and analyze the genome and transcriptome of the newly isolated *T. terrestris* strain LPH172, which is characterized by superior enzymatic activity, thermostability, and pH stability [17]. Our current study aimed to elucidate the lignocellulose-degrading machinery of the fungus in detail and identify novel carbohydrate-active enzymes (CAZymes). We observed some genomic differences between LPH172 and the previously sequenced strain NRRL 8126. To corroborate genomic CAZyme analysis with transcriptome data, we grew the fungus on four substrates: glucose, Avicel, rice straw, and beechwood xylan. We observed that the fungus relied mainly on LPMOs and canonical cellulases when grown on cellulosic substrates and on hemicellulosic substrates, more conventional hemicellulases were induced together with some LPMOs. Interestingly, we also report co-expression and co-upregulation between LPMOs and other AA enzymes.

## Results

### Strain identification

We previously isolated the *T. terrestris* strain LPH172 from compost in Northern Vietnam and showed that it could be exploited as an industrially relevant enzyme producer [17]. To confirm the identity of the fungus, first, we used two common fungal housekeeping genes [38] encoding transcription elongation factor 1-*α* and *β*-tubulin. The homologous gene sequences used for the identification procedure are listed in Additional file 1. Both housekeeping genes were 100% identical (e-value 0) to the *T. terrestris* NRRL 8126 homologues.

Furthermore, we used phylogenetic analysis of closely related species to confirm the strain identity. Three maximum likelihood phylogenetic trees were constructed based on the internal transcribed spacer (*ITS-D1D2*), RNA polymerase II (*RPB2*) and *β*-tubulin (*TUB2*) genes. In all three constructed trees, the *T. terrestris* strain LPH172 clustered closely together with four other *T. terrestris* strains (Additional file 1: Figure S1).

Both of these results confirmed the fungus in the current study to be a strain of *T. terrestris.*

### Growth on different carbohydrates

**Fig. 1:**
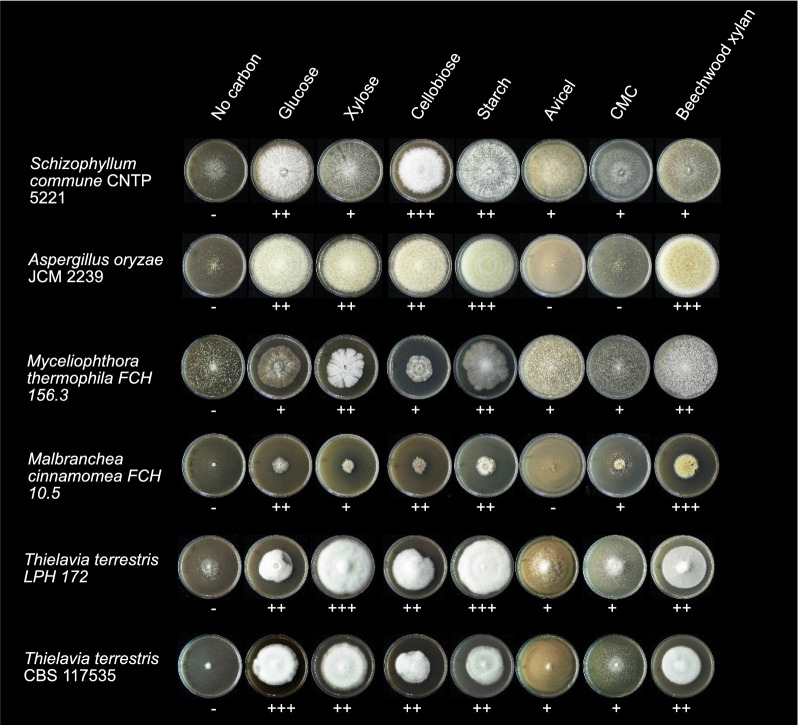
Growth of *T. terrestris* LPH172 and other biomass-degrading filamentous fungi on different carbon sources. Seven different carbohydrate substrates at 2% (w/v) were used as sole carbon sources for growth on agar plates: monosaccharides (glucose, xylose), disaccharides (cellobiose), and polysaccharides (starch, Avicel, carboxymethyl cellulose—CMC, beechwood xylan). No carbon source was added in the control. The plates were incubated at 30 °C (*S. commune*, *A. oryzae*) or 50 °C (*M. thermophila*, *M. cinnamomea*, *T. terrestris*) for 2–7 days. Growth was evaluated visually and categorized from − (no growth) to +++ (very good growth), depending on the extent and density of the mycelium

To assess the ability of *T. terrestris* LPH172 to utilize different carbon sources, the strain was grown on various defined substrates on agar. Growth was measured by the diameter and density of mycelia and was compared to a selection of known mesophilic and thermophilic biomass degraders (Fig. 1). *T. terrestris* LPH172 grew best on starch and xylose, followed by glucose, cellobiose, and beechwood xylan, whereas only modest growth was observed on the cellulosic substrates Avicel and carboxymethyl cellulose (CMC). This finding suggests relatively high activity of amylases, xylanases, and *β*-glucosidases. Direct comparison to the previously sequenced *T. terrestris* CBS 117535 (GenBank acc. nr PRJNA249224) showed that LPH172 grew slightly better on most substrates except glucose. Good growth was observed on pectin and inulin (a fructose-based polymer), whereas growth on locust bean gum and guar gum (galactomannans), as well as bark powder was poor (Additional file 2).

### Genome characterization

#### General features

The genome of *T. terrestris* LPH172 was sequenced on a PacBio RS II instrument by GATC Biotech (Constance, Germany); it yielded 5,27,523 reads comprising over 7 billion bases. Table 1 gives an overview of the sequenced *T. terrestris* LPH172 genome. Its size was determined to be 36.6 Mb and guanine and cytosine (GC) content was 54.80%. Assembly quality, based on basic sequence statistics, was high as revealed by an average contig size (N50) of 3 Mb and N50 read length of 19,832. To assess completeness and integrity of the genome assembly, Benchmarking Universal Single-Copy Orthologs (BUSCO) analysis was performed [39]. Over 98% of BUSCO genes in the LPH172 genome were complete, indicating excellent assembly integrity. Gene prediction algorithms identified 10,128 protein-coding genes.

**Table 1:** Overview of *T. terrestris* LPH172 genome as sequenced in this study

Genome assembly	
Number of nucleotides	36,579,697
GC content	54.80%
N50 (bp)	3,006,457
Number of protein-coding genes	10,128
Average gene length (bp)	1628
Average CDS length (bp)	1355
Average number of exons per gene	3
Average exon length (bp)	460
Longest genes (bp)	25,297
Longest exons (bp)	9745
Shortest genes (bp)	39
Shortest exons (bp)	3
Fraction of genome covered by	
Genes	45.10%
Exons	37.80%
Introns	7.30%
Gene annotation	
Genes with functional annotation	8879
Genes without functional annotation	1249
Genes annotated (BLASTp, e-value <1e^−6^)	6114

The size of fungal genomes can vary by orders of magnitude and the average for Ascomycota is 36.91 Mb [40, 41]. Table 2 gives a brief overview of the characteristics of the LPH172 genome compared to other industrial and lignocellulose-degrading fungi with varied origin and thermostability. Even though the genome of *T. terrestris* NRRL 8126 (GenBank assembly nr GCA_000226115), sequenced in 2010, was slightly larger than that of LPH172, our analysis suggested LPH172 contained approximately 200 more genes. This discrepancy, in addition to inherent differences between the two strains, is likely a consequence of ongoing improvements in sequencing and annotation. The genome size of LPH172 was similar to those of other fungi listed in Table 2, as well as to the average Ascomycota genome. The same was true for the average genome GC content. The average gene length in strain LPH172 was 1628 bp and the average coding sequence was 1355 bp (Table 1). On average, three exons per gene were predicted, with exons covering 45.10% of the genome. 88% of the genes could be functionally annotated with BLASTp, 69% of which with high certainty (e-value <1e^−6^).

**Table 2:** Genome characteristics in *T. terrestris* LPH172 and different industrial and lignocellulose-degrading fungi

Organism	Phylum	Thermotolerance	Genome (Mb)	Genome GC (%)	Number of protein-coding genes	Source
*A. oryzae*	Ascomycota	Mesophilic	37.9	47.2	12,030	[42]
*M. thermophila*		Thermophilic	38.7	51.4	9110	[25]
*M. cinnamomea*		Thermophilic	25.0	49.8	9437	[43]
*P. anserina*		Mesophilic	35.0	52.0	10,588	[44]
*T. terrestris* LPH172		Thermophilic	36.6	54.8	10,128	This article
*T. terrestis* NRRL 8126		Thermophilic	36.9	54.7	9813	[25]
*G. trabeum*	Basidiomycota	Mesophilic	37.2	52.9	11,755	[45]
*S. commune*		Mesophilic	38.5	N/A	13,210*	[46]
*R. pusillus*	Zygomycota	Thermophilic	25.6	45.0	10,898	[47]
*R. oryzae*		Mesophilic	39.1	35.4	13,895	[48]

*nr of genes

#### Thermostability features

Although there is no clear consensus on the causes contributing to elevated optimum growth temperatures and thermotolerance in fungi, possible factors include a reduction in genome size [49], higher average GC content in coding regions, and greater GC content in the third position of codons (GC3 content) [25, 50].

In contrast to the thermophilic Ascomycete *Malbranchea cinnamomea* [43], the genome of *T. terrestris* LPH172 was not smaller compared to that of other mesophilic fungi (Table 2). Normalized (gene length) GC content in gene-coding sequences was 63.5%, which was higher than the genome average of 54.80%. When looking only at the subset of genes encoding CAZymes, the average normalized GC content was even higher (64.5%). Normalized GC3 content in LPH172 was also high, amounting to 80.7% in coding sequences and 85.7% in CAZyme-encoding sequences. We also detected gene TT_05393, encoding an unknown protein with 33% identity (e-value 1.3e^−19^) to the known thermotolerance gene *THTA* from *Aspergillus fumigatus* (GenBank: AY560012.1) [51].

### CAZyme comparison with other fungi

**Fig. 2:**
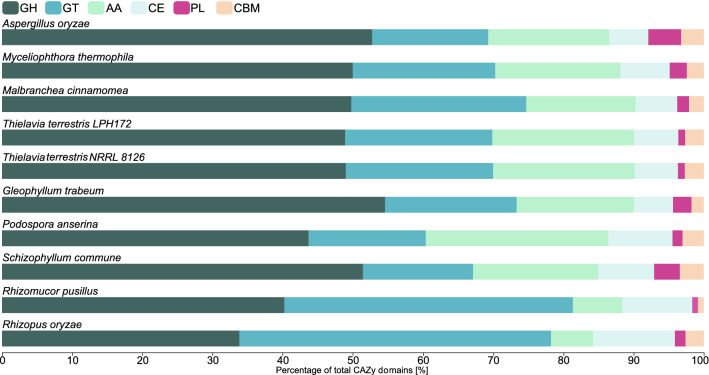
Relative numbers of CAZy domains from six CAZy families in various filamentous fungi. For each species, the numbers of predicted CAZyme domains were normalized to the total number of predicted CAZyme domains. GH, glycoside hydrolase; GT, glycoside transferase; AA, auxiliary activity; CE, carbohydrate esterase; PL, polysaccharide lyase; CBM, carbohydrate-binding module. Predictions were made with dbCAN2 (HMMER algorithm). CE10 family domains were excluded

**Fig. 3:**
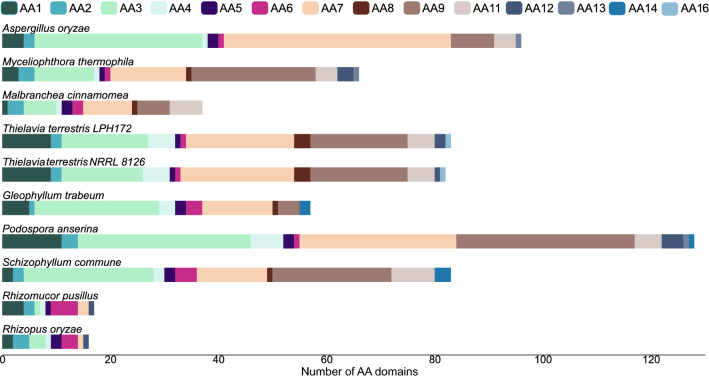
Number of auxiliary activity (AA) CAZyme family domains in different filamentous fungi. For each species, the number of predicted AA domains categorized into AA families 1–16 is shown. Predictions were made with dbCAN2 (HMMER algorithm)

Plant biomass-degrading and other CAZymes are catalogued into classes, families and subfamilies in the Carbohydrate Active enZymes (CAZy) database (http://www.cazy.org/) [52].The number of individual CAZyme domains and distribution across different CAZy families in *T. terrestris* LPH172 was analyzed and compared to other known fungal biomass degraders to assess the propensity for lignocellulose degradation (Table 3). In total, 411 individual CAZy domains were detected in LPH172 using dbCAN2 (HMMER algorithm) (Additional file 3). Most CAZy domains were found to be GHs (201 candidates), with GH18 (*n* = 15), GH16 (*n *= 14), GH3 (*n* = 12), and GH47 (*n *= 10) being the most abundant families. There were also 86 glycosyl transferase (GT) domains, 4 polysaccharide lyase (PL) domains, 26 carbohydrate esterase (CE) domains, 83 AA domains, and 11 carbohydrate-binding module (CBM) domains. Compared to strain NRRL 8126, two more GH (one GH16 and one GH47) domains were identified in LPH172, as well as one additional AA12, one GT2, and one CE1 domain (Additional file 3). *T. terrestris* LPH172 had a relatively low number of PL domains compared to other fungi (Fig. 2), but a larger complement of AA family domains, particularly AA7 (*n *= 20), AA9 (*n *= 18) and AA3 (*n *= 16) (Fig. 3). Five members of AA11 (chitin-cleaving) LPMO domains were detected in both *T. terrestris* strains, but no AA13 (starch-cleaving LPMOs) or AA14 (xylan-cleaving LPMOs) domains were observed. LPH172 and NRRL 8126 were the only fungi, among the ones selected, presenting an AA16, a recently characterized C1-hydroxylating LPMO [53].

**Table 3:** Comparison of the number of individual CAZy domains in *T. terrestris* LPH172 and other filamentous fungi

	GH	GT	PL	CE	AA	CBM	Total
*Aspergillus oryzae*	292	92	26	31	96	18	555
*Myceliophthora thermophila*	185	75	9	26	66	9	370
*Malbranchea cinnamomea*	118	59	4	14	37	5	237
*Thielavia terrestris* LPH172	201	86	4	26	83	11	411
*Thielavia terrestis* NRRL 8126	199	85	4	25	82	11	406
*Gloeophyllum trabeum*	186	64	9	19	57	6	341
*Podospora anserina*	215	82	7	45	128	15	492
*Schizophyllum commune*	239	73	17	37	83	16	465
*Rhizomucor pusillus*	97	99	2	24	17	2	241
*Rhizopus oryzae*	90	118	4	31	16	7	266

All CAZy domains were identified using dbCAN2 (HMMER algorithm)

CE10 family domains were excluded

*GH* glycoside hydrolase, *GT* glycoside transferase, *AA* auxiliary activity, *CE* carbohydrate esterase, *PL* polysaccharide lyase, *CBM* carbohydrate-binding module

Putative candidates for CAZymes capable of degrading all major lignocellulosic polymers (cellulose, xylan, xyloglucan, (galacto)glucomannan, pectin, and lignin), as well as starch, inulin, and chitin were found. This finding was in line with growth of *T. terrestris* on all these carbon sources (Additional file 2). At least ten putative homologues of known transcription factors directly regulating (hemi)cellulose utilization in ascomycetous fungi [54] were also detected in the genome of *T. terrestris* LPH172 (Additional file 4).

#### Transcriptome analysis

##### Highly expressed CAZyme-encoding genes during growth on Avicel, rice straw, and beechwood xylan

To verify the genome annotation of LPH172 and analyze gene expression, in particular of CAZyme-encoding genes, the transcriptome was analyzed under different growth conditions. The fungus was grown in shake flasks on four substrates—glucose, Avicel, rice straw and beechwood xylan—and total mRNA was extracted and sequenced. Glucose was chosen as reference monosaccharide because its degradation involves a limited number of CAZymes and should, therefore, reflect expression of mostly constitutive CAZyme-encoding genes. Beechwood xylan, comprising a xylan backbone with 4-*O*-methyl glucuronic acid side groups, was selected to detect CAZymes required for hardwood hemicellulose degradation [43]. Rice straw, which contains approximately 12% lignin, 38% cellulose, and 25% hemicellulose [55] was chosen to represent a complex, heterogeneous substrate requiring a large array of different CAZymes for degradation. Importantly, rice straw has also vast potential as feedstock in biorefinery applications. Finally, Avicel, which is up to 98% cellulose [56, 57], was selected to identify enzymes required to degrade a highly crystalline and recalcitrant cellulosic substrate. Initially, our transcriptome analysis also included corn cob xylan as an arabinoxylan-containing substrate model for cereals. The results were not included because the purchased corn cob xylan turned out to be composed only of xylo-oligomers [58]. Transcriptome data from RNAseq was used to refine gene annotation through *ab initio* training with GeneMark v4.3 and an evidence-guided build with MAKER package v3.01.1. Results are summarized in Tables 4–6.

**Table 4:** Twenty most highly expressed CAZyme-encoding genes during growth of *T. terrestris* LPH172 on Avicel

Transcript ID	TPM	CAZy domain(s)	Putative function
TT_05546	12001	GH7-CBM1	Exoglucanase
TT_07456	8862	AA9	LPMO
TT_06655	8643	GH6	1,4-*β*-d-Glucan cellobiohydrolase
TT_08370	8271	AA9	LPMO
TT_06499	6276	CBM1	Feruloyl esterase
TT_00224	4790	GH5_5	Endoglucanase
TT_05797	4654	GH7-CBM1	Endoglucanase
TT_03075	4576	GH11-CBM1	Endo-1,4-*β*-xylanase
TT_04350	4537	AA9-CBM1	LPMO
TT_07210	3659	GH30_7	Glucosylceramidase
TT_08166	3512	CE5-CBM1	Acetylxylan esterase
TT_06407	3218	AA9	LPMO
TT_09000	3188	GH45	Endoglucanase
TT_01736	2677	AA9-CBM1	LPMO
TT_01019	2322	GH5_5	Endoglucanase
TT_04380	1599	AA3_1-AA8	Cellobiose dehydrogenase
TT_01839	1429	GH11	Endo-1,4-*β*-xylanase
TT_08161	1335	GH10-CBM1	Endo-1,4-*β*-xylanase
TT_03756	1104	GH72	1,3-*β*-Glucanosyltransferase
TT_01913	919	GH132	Secreted *β*-glucosidase

TPM values indicate average normalized transcript levels from three biological replicates. CAZy domains were predicted by dbCAN2 and functions were annotated by BLASTp search against the UniProt/Swiss-Prot reference dataset

To identify which CAZyme-encoding genes were the most highly expressed (by transcript abundance) on each chosen substrate, we looked at the top 20 (arbitrary number) candidates under each growth condition, ranked by their average transcripts per million (TPM) value. The complete list of all expressed genes with their TPM values is available in Additional file 5.

Among the top 20 highly expressed CAZyme-encoding genes on Avicel (Table 4) there were in total 11 putative cellulase and LPMO-encoding genes—six cellulases and five LPMOs. These highly expressed cellulase genes included one encoding a putative GH7-CBM1 exoglucanase (TT_05546), a GH6 cellobiohydrolase (TT_06655) and four endoglucanases GH7-CBM1 (TT05797), GH5_5 (TT01019 and TT_00224) and GH45 (TT_09000). All five highly expressed LPMO-encoding genes (TT_07456, TT_08370, TT_04350, TT_06407, TT_01736) belonged to the AA9 family. A gene encoding a CAZyme possibly acting in synergy with LPMOs was also highly expressed—a putative AA3-AA8 cellobiose dehydrogenase encoding gene (TT_04380). Genes encoding five putative xylan-active enzyme genes were also highly abundant on Avicel, encoding a CE5-CBM1 acetylxylan esterase (TT_08166), a feruloyl esterase with a CBM1 domain (TT_06499), two GH11 endo-*β*-1,4-xylanases (TT_03075 and TT_01839) and a GH10-CBM1 endo-*β*-1,4-xylanase (TT_08161). In general, seven of the 20 highly expressed CAZyme genes on Avicel also included a cellulose-binding CBM1 domain.

When the fungus was cultivated on rice straw (Table 5), only one putative cellulase gene (GH131 endoglucanase; TT_01081) was among the top 20 most highly expressed CAZyme-encoding genes. However, five AA9 LPMOs were again highly expressed (TT_07456, TT_08370, TT_04352, TT_06407, TT_03770) - of which TT_07456, TT_08370 and TT_06407 were among the top 20 highly expressed CAZyme genes also on Avicel. None of these putative LPMO-encoding genes expressed during growth on rice straw included a CBM1. For possible xylan backbone degradation, a putative GH11 endo-*β*-1,4-xylanase (TT_01839) encoding gene was highly expressed and a putative GH11-CBM1 endo-*β*-1,4-xylanase (TT_03075) as well. Both were also highly expressed on Avicel. In addition, a gene encoding a putative CE16 acetyl esterase (TT_06012) possibly cleaving acetyl groups from xylan was among the highly expressed CAZyme genes. Interestingly, on rice straw, genes encoding putative mannosyltransferases belonging to the CAZy families GT15 (TT_07992 and TT_03483), GT32 (TT_08079) and GT2 (TT_05489) were also among the top 20 most highly expressed CAZyme-encoding genes. Moreover, a CE5-CBM1 cutinase (TT_08797) was among these top 20 expressed CAZyme genes on rice straw, possibly active on plant cuticle.

**Table 5:** Twenty most highly expressed CAZyme-encoding genes during growth of *T. terrestris* LPH172 on rice straw

Transcript ID	TPM	CAZy domain(s)	Putative function
TT_01839	8312	GH11	Endo-1,4-*β*-xylanase
TT_07456	963	AA9	LPMO
TT_08370	462	AA9	LPMO
TT_07992	418	GT15	Glycolipid 2-*α*-mannosyltransferase
TT_08797	400	CE5	Cutinase
TT_08097	276	GT32	Initiation-specific *α*-1,6-mannosyltransferase
TT_05010	249	GH25	*N,O*-Diacetylmuramidase
TT_01081	241	GH131	Endoglucanase
TT_04352	234	AA9	LPMO
TT_05489	178	GT2	Dolichol-phosphate mannosyltransferase subunit
TT_03075	171	GH11-CBM1	Endo-1,4-*β*-xylanase
TT_06407	167	AA9	LPMO
TT_08740	166	CBM52	Uncharacterized secreted protein
TT_09851	162	GH128	Alkali-sensitive linkage protein
TT_03483	158	GT15	Mannosyltransferase
TT_05929	139	GT31	*β*-1,3-*N*-Acetylglucosaminyltransferase
TT_03756	135	GH72	1,3-*β*-Glucanosyltransferase
TT_06012	125	CE16	Acetylesterase
TT_01913	119	GH132	Secreted *β*-glucosidase
TT_03770	117	AA9	LPMO

TPM values indicate average normalized transcript levels from three biological replicates. CAZy domains were predicted by dbCAN2 and functions were annotated by BLASTp search against the UniProt/Swiss-Prot reference dataset

When grown on beechwood xylan, curiously, only two putative xylanase encoding genes were among the 20 most highly expressed genes coding for CAZymes (Table 6). These encoded a putative CE5-CBM1 acetylxylan esterase (TT_08166) and a GH11 endo-*β*-1,4-xylanase (TT_01839), the last one also highly expressed on Avicel and rice straw. No AA9 LPMO-encoding genes were among the 20 most highly expressed CAZyme genes on beechwood xylan, though, a gene encoding a putative AA11 LPMO (TT_09754) was among the top 20 highly expressed genes. Interestingly, several genes encoding putative transferase genes were also highly expressed on beechwood xylan, like on rice straw. These included two genes encoding putative GH72 *β*-1,3-glucanosyltransferases (TT_03756 and TT_03035) and a gene encoding a putative GT32 mannosyltransferase. Genes coding for putative GH16 glucosidases were also among the top 20 most abundant transcripts on beechwood xylan (TT_08846, TT_09509 and TT_02410). Interestingly, also genes encoding a putative GH37 trehalase (TT_00335) and a putative AA2 catalase-peroxidase (TT_08899) were highly expressed on this hemicellulosic substrate. During growth on both rice straw and beechwood xylan, a gene encoding a putative CAZyme with a CBM52 domain was highly expressed (TT_08740). A putative GH72 *β*-1,3-glucanosyltransferase TT_03756 was also among the most highly expresses genes on all three substrates.

**Table 6:** Twenty most highly expressed CAZyme-encoding genes during growth of *T. terrestris* LPH172 on beechwood xylan

Transcript ID	TPM	CAZy domain(s)	Putative function
TT_05010	2452	GH25	*N,O*-Diacetylmuramidase
TT_01913	1466	GH132	Secreted *β*-glucosidase
TT_03756	1366	GH72	1,3-*β*-Glucanosyltransferase
TT_09441	1359	CE9	*N*-Acetylglucosamine-6-phosphate deacetylase
TT_08166	1329	CE5-CBM1	Acetylxylan esterase
TT_08780	903	GH17	Glucan endo-1,3-*β*-glucosidase
TT_03035	892	GH72	1,3-*β*-Glucanosyltransferase
TT_08846	743	GH16-CBM18	Glycosidase
TT_09754	736	AA11	LPMO
TT_01404	608	GH17	*β*-Glucosidase
TT_00335	516	GH37	Trehalase
TT_09509	487	GH16	Glycosidase
TT_06210	471	GH30_3	Endo-1,6-*β*-d-glucanase
TT_08740	469	CBM52	Uncharacterized secreted protein
TT_00639	459	GH20	*β*-Hexosaminidase
TT_01839	419	GH11	Endo-1,4-*β*-xylanase
TT_08097	383	GT32	Initiation-specific *α*-1,6-mannosyltransferase
TT_08899	375	AA2	Catalase-peroxidase
TT_05769	317	GH128	Alkali-sensitive linkage protein
TT_02410	310	GH16	Extracellular glycosidase

TPM values indicate average normalized transcript levels from three biological replicates. CAZy domains were predicted by dbCAN2 and functions were annotated by BLASTp search against the UniProt/Swiss-Prot reference dataset

##### Upregulated CAZyme-encoding genes on Avicel, rice straw, and beechwood xylan

**Fig. 4:**
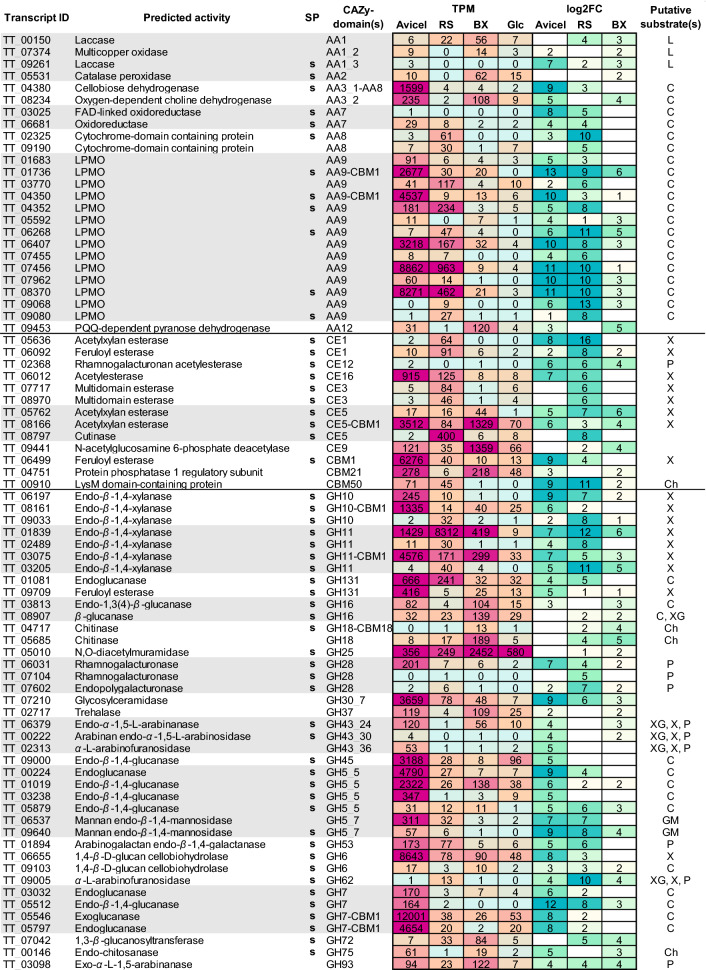
Combined expression and upregulation values of CAZyme-encoding genes during cultivation on four different substrates. Putative CAZyme-encoding genes involved in biomass degradation were analyzed for their expression levels (TPM, transcripts per million) from three biological replicates, as well as their differential expression (log2FC). The heatmap shows a combination of top forty most highly upregulated CAZyme-encoding genes on three substrates Avicel, rice straw (RS), beechwood xylan (BX) when compared to glucose (Glc). Shading ranges from low expression (light blue) to high expression (magenta). Log2 fold-change (log2FC) shows gene expression during cultivation on Avicel, RS, and BX compared to cultivation on glucose. Shading of upregulated genes (i.e., gene transcripts more abundant on Avicel, RS, and/or BX than on glucose) ranges from light yellow (low upregulation) to dark green (high upregulation). Downregulated genes or genes for which no differential expression was detected or where upregulation was not significant are indicated by blank cells. Only significantly upregulated genes are shown (*p* ≤ 0.05). All numbers were rounded to the nearest integer. The putative activities of the gene products are based on BLASTp predictions. CAZy domains were analyzed with dbCAN2. The presence of putative signal peptides (SP), predicted by SignalP 4.0, is indicated by a small s. Putative substrates of the CAZymes are: C, cellulose; Ch, chitin; GM, glucomannan; L, lignin; P, pectin; S, starch; X, xylan; XG, xyloglucan

Absolute gene expression levels do not show the full spectrum of available lignocellulose-degrading enzymes in the organism, because many enzymes are sufficiently active at low concentration and some are constitutively expressed. Therefore, to understand which genes were induced under the tested conditions (Avicel, rice straw or beechwood xylan), we investigated the differential expression of CAZyme-encoding genes with respect to glucose as reference using edgeR [59]. We focused on the transcripts that were significantly highly expressed (i.e., upregulated) compared to glucose in the test conditions (*p* ≤ 0.05) (Additional file 6). A combined list of the top 40 most highly upregulated CAZyme-encoding genes on all three substrates is shown in Fig. 4.

On Avicel, the most highly upregulated genes encoded a putative AA9-CBM1 LPMO gene, (TT_01736, log_2_ fold-change (log2FC) 13). Other putative AA9 LPMO-encoding genes were also highly upregulated: TT08370 and TT07456 had log2FC 11 and TT04350, TT07962 and TT6407 all had log2FC 10. A gene encoding a putative AA3-AA8 cellobiose dehydrogenase was also among the most highly upregulated (TT04380, log2FC 9). All AA9 (besides TT07962) and the AA3-AA8 CAZyme-encoding genes presented also very high TPM values, indicating both high upregulation and high expression levels. Other putative CAZyme genes highly upregulated on Avicel encoded mainly GH7 (TT05797 with log2FC 8, TT05512 with log2FC 12, TT03032 with log2FC 6, TT05546 with log2FC 8), GH5 (TT01019 with log2FC 6, TT03238 with log2FC 4, TT00224 with log2FC 9) and GH45 (TT09000 with log2FC 5) endo- and exo-glucanases. One putative GH6 cellobiohydrolase encoding gene was also highly upregulated (TT06655, log2FC 8). Interestingly, numerous non-cellulose acting CAZyme-encoding genes were also upregulated on Avicel. These included two feruloyl esterases (TT_06499 with log2FC 9 and TT_09709 with log2FC 5), five GH10 and GH11 xylanases (TT_06197 with log2FC 9, TT_03205 with log2FC 5, TT_03075 with log2FC 7, TT_01839 with log2FC 7, TT_08161 with log2FC 6), a CE1 acetylxylan esterase (TT_05636 with log2FC 8), a CE12 rhamnogalacturonan acetylesterase (TT_02368 with log2FC 6), a CE16 acetylesterase (TT_06012 with log2FC 7) and CE5-CBM1 a acetylxylan esterase (TT_08166 with log2FC 6). Two putative AA7 oxidoreductase encoding genes were highly upregulated on Avicel (TT_03025 with log2FC 8 and TT_06681 with log2FC 4). Two GH5_7 mannan endo-*β*-1,4-mannosidase (TT09640 and TT06537) encoding genes were also greatly upregulated on this substrate.

On rice straw, the most highly upregulated genes encoded a putative CE1 acetylxylan esterase (TT_05636 with log2FC 16), an AA9 LPMO (TT_09068 with log2FC 13) and a GH11 endo-*β*-1,4-xylanase (TT_01839 with log2FC 12). In general, the set of forty most highly upregulated putative CAZyme-encoding genes on rice straw shared some candidates with Avicel, such as nine AA9 LPMO genes and four hemicellulose-active GH10 and GH11 xylanases, a GH7 endo-*β*-1,4-glucanase and CE1, CE5 and CE16 esterases. A putative gene encoding a CBM50 domain (TT_00910) was highly upregulated both on Avicel and rice straw. In addition to the AA9 LPMO genes highly upregulated on Avicel as well, on rice straw two more AA9 genes appeared: TT_03770 and TT_07455 (both with log2FC 6). Generally, more hemicellulose-active enzyme encoding genes were among the forty highly upregulated genes on rice straw than on Avicel, which is expected with the more complex substrate composition of rice straw. These genes encoded putative GH10 (TT_09033 with log2FC 8) and GH11 (TT_02489 with log2FC 8) endo-*β*-1,4-xylanases, a GH62 *α*-l-arabinofuranosidase (TT_09005 with log2FC 10), two CE3 esterases (TT_07717 and TT_08970, both with log2FC 6), a CE1 feruloyl esterase (TT_06092 with log2FC 8), a CE5 acetylxylan esterase (TT_05762 with log2FC 7) and a CE5 cutinase (TT_08797 with log2FC 8). The two putative mannosidase encoding genes highly upregulated on Avicel (TT_09640 with log2FC 8 and TT_06537 with log2FC 7) were also highly upregulated on rice straw. Two putative AA7 oxidoreductase encoding genes (TT_06681 with log2FC 4 and TT_03025 with log2FC 5), as well as a transcript with an AA8 domain (TT_02325 with log2FC 10), were also among the top forty highly upregulated genes on rice straw.

On beechwood xylan, upregulation of CAZyme-encoding genes was more subdued with generally lower log2FC values, and fewer overlaps with the other two substrates were detected. Interestingly, the second most highly upregulated gene encoded a putative AA9-CBM1 LPMO (TT_01736 with log2FC 6). Transcripts of six other AA9 LPMOs were also more abundant on beechwood xylan compared to glucose (TT_06268, log2FC 5; TT_07962, log2FC 3; TT_08370, log2FC 3; TT_06407, log2FC 3; TT_05592, log2FC 3 and TT_09068, log2FC 3), although not as strongly upregulated as on other substrates. A gene encoding a putative AA12 PQQ-dependent pyranose dehydrogenase (TT_09453, log2FC 5) was also among the top highly upregulated genes on BX. The same applies for a putative AA3_2 dehydrogenase TT_08234 with log2FC 4.

Despite beechwood xylan being a pure xylan substrate, only a fraction of upregulated CAZyme genes were encoding putative xylan-acting enzymes, such as CE5 acetylxylan esterases TT_05762 (log2FC 6), TT_05762 (log2FC 6) and TT_08166 (log2FC 4), GH11 endo-1,4-*β*-xylanases TT_01839 (log2FC 6), TT_03205 (log2FC 5) and TT_03075 (log2FC 3). Interestingly, no GH10 endo-1,4-*β*-xylanases nor CE1, CE3 or CE16 esterase encoding genes were among the most highly upregulated genes on BX. Two genes encoding enzymes that are active on chitin and possibly involved in fungal cell wall modulation were also highly upregulated on beechwood xylan, such as GH18 chitinases TT_05685 (log2FC 5) and TT_04717 (log2FC 4). A variety of cellulose-, pectin- and arabinan-active CAZyme-encoding genes were upregulated at low levels (log2FC 2-4); the same was observed for some genes encoding enzymes typically associated with lignin degradation (Fig. 4, Additional file 6).

## Discussion

The present study sought to explain in detail the enzymatic machinery *T. terrestris* LPH172 possesses to break down major lignocellulosic polymers based on genome and transcriptome analysis. Specifically, cellulose degradation appeared to rely mostly on LPMOs and some highly expressed canonical cellulases. Compared to other carbon sources, growth on Avicel was poor, yet LPH172 performed better on this substrate than most other fungi (Fig. 1). Poor growth on Avicel could result from lack of cellulase induction or the high crystallinity of Avicel. We noticed similar discrepancy between putative cellulose-degrading genes and poor growth on Avicel in our previous work with *M. cinnamomea* [43]. Growth discrepancies between the two *T. terrestris* strains LPH172 and CBS 117535 corroborate previously reported differences in biomass degradation and enzyme production between strains of the same species [17]. It has been reported that two strains of *A. niger*, for example, produced diverse sets of biomass-degrading enzymes, even when grown on the same plant biomass substrates [37, 60]. This was mainly attributed to the postgenomic and regulatory differences between strains. Differential gene expression analysis helped to suggest the main enzymes involved in degradation of tested substrates (Fig. 4). The range of upregulated CAZyme-encoding genes was perhaps more diverse than expected, with genes encoding mannanases, xylanases, pectinases and lignin-active enzymes being upregulated on all substrates regardless of the presence or absence of the corresponding polymers. Co-regulation of biomass-degrading enzymes or the presence of traces of other polymers could explain induction of these genes. Enzymological studies that compare the activities and activity optima of these enzymes will help determine the function of seemingly redundant enzymes such as the abundantly expressed AA9 LPMO genes.

(Hemi)cellulase encoding genes were highly expressed and upregulated on Avicel, which is a cellulosic substrate and, hence, should not require hemicellulases for degradation. However, this type of unanticipated expression has been shown before in *T. terrestris*, when the cellulosic substrate CMC induced xylanase production [16]. On the other hand, minor amounts of xylan previously reported to be found in Avicel [56, 57] could also stimulate xylanase expression. Putative direct (hemi)cellulase regulating transcription factors were analyzed in the genome of LPH172 and the presence of the prominent promiscuous regulator Xyr1 known to affect both cellulases and hemicellulases [61, 62] was detected (Additional file 4). This finding supports the hypothesis of (hemi)cellulase co-regulation in this fungus or alternatively, a combination of co-regulation and induction due to the xylan contamination in Avicel.

Since their discovery a decade ago, LPMOs have been studied in several different fungal, bacterial and even insect species, with new families and activities being continuously reported [53, 63, 64]. In *T. terrestris* LPH172, the transcriptomic data indicate that AA9 LPMOs play a crucial role in cellulose degradation, as six such enzymes were highly upregulated and five were very highly expressed during growth on Avicel. Interestingly, on all three polymeric substrates the highest upregulated genes belonged to this family. Several AA9 genes in LPH172 were highly upregulated on rice straw, which contains some cellulose, but also on purified beechwood xylan. We hypothesize that traces of cellulose in the beechwood xylan substrate induce the expression of cellulose-degrading enzymes, or that co-regulation occurs. Alternatively, certain AA9 LPMOs could act on non-cellulosic substrates, including xylan, mannan or xyloglucan, as reported, for instance, for AA9 LPMOs from *M. cinnamomea* [65] and *M. thermophila* [66] and *N. crassa* [67]. A clear preference for CBM1-containing genes was shown among the highly expressed and upregulated CAZyme genes on Avicel but also on rice straw, supporting the predicted cellulose-binding character of CBM1 proteins.

Other members of AA CAZy families were also highly expressed and/or significantly upregulated during growth of LPH172 on various substrates. AA3_1-AA8 cellobiose dehydrogenases (CBD) act as reducing agents to fuel LPMO reactions ^[[23, 28, 68–71]]^. However, not all fungi containing LPMO genes contain supplementary cellobiose dehydrogenase encoding genes [68]. We observed high co-expression and co-upregulation of these enzyme encoding genes on cellulose-containing substrates. The AA3_1-AA8 CBD gene (TT_04380) that was highly co-upregulated with several AA9 LPMOs in our study has been shown to act in synergy with a *Thermoascus aurantiacus* GH61A (AA9) enzyme [28]. Interestingly, on the cellulosic substrates we also noted the upregulation of two AA8 cytochrome domain containing CAZyme genes (TT_02325 and TT_09190) which could also potentially reduce the copper in the active center of LPMOs. However, the electron transfer to these AA8 domains remains unclear. According to Pfam analysis, TT_09190 also contains a putative sugar transporter domain. Moreover, absence of AA8 co-upregulation with AA9 LPMO genes on beechwood xylan might indicate that a different reduction system is utilized on hemicellulosic substrates than on the cellulosic substrates. AA3_2 single-domain flavoenzymes have also been shown to act in synergy as electron donors for LPMOs [72]. Here, we detected co-expression and co-upregulation of an AA3_2 dehydrogenase gene (TT_08234) and AA9 genes on Avicel and beechwood xylan. Other AA CAZymes capable of producing H_2_O_2_, and therefore potentially serving as LPMO co-factors, are AA7 family oxidoreductases. In fact, an AA7 enzyme with a novel oligosaccharide dehydrogenase activity has been recently shown to both transfer electrons to the LPMO active site copper but also produce H_2_O_2_ as a co-substrate of LPMOs [72]. Here, AA7 encoding genes TT_06681 and TT_03025 were upregulated on both Avicel and rice straw; however, their exact roles in biomass degradation have to be elucidated by future studies. Interestingly, out of the 20 AA7 domains in this strain only four were expressed according to our transcriptomic analysis. An AA3 enzyme (TT_07514) that did not fit in the top genes mentioned in our analysis in Fig. 4 but was still significantly upregulated on all substrates, is not yet classified into an AA3 sub-family according to dbCAN. This AA3 encoding gene found to contain two putative GMC-oxidoreductase domains using Pfam analysis, as well as a putative bacterial luciferase-like domain. To our knowledge, such a domain has not been seen before in combination with AA3 domains and may indicate a fifth sub-family of AA3 CAZymes, but further studies are crucial to substantiate this hypothesis.

In general, the elevated number of LPMO-encoding genes in the fungus, together with their high expression and upregulation confirm the importance of (AA9) LPMOs for plant biomass decomposition by *T. terrestris* and explains why studying the secretomes of this species had such a clear cellulase-boosting effect [19]. The numerous LPMOs in filamentous fungi support the concept of microbial mutualism. According to this concept, some fungi are responsible mainly for LPMO secretion and for attacking crystalline substrate surfaces, thereby making way for others to degrade amorphous polysaccharides and eventually benefitting the whole microbial community [60, 73]. Such interactions have been documented with regard to the mutually beneficial synthesis of vital growth substances in fungi [74]. Analogously, white rot fungi are known to degrade lignin, whereas brown rotters mainly modify lignin [7], indicating unique specifications for lignocellulose degradation in different filamentous fungi.

Finally, regarding possible genetic factors contributing to fungal thermostability [50], the genome of *T. terrestris* LPH172 revealed high GC content in the coding sequences of all genes and in those encoding CAZymes as well. In addition, the observed high GC3 content could contribute to the thermophilic lifestyle in *T. terrestris,* as also noted by Berka et al. [25]. Further research is needed to confirm and elucidate the mechanisms of this interesting phenomenon.

## Conclusion

We sequenced and analyzed the genome of a novel *T. terrestris* strain LPH172. Both genome and transcriptome analyses of the novel thermophilic *T. terrestris* strain LPH172 revealed in detail the enzymatic machinery used by the fungus to break down lignocellulosic biomass. Using transcriptome data from growth on glucose, Avicel, rice straw, and beechwood xylan we conclude that the fungus relies on an LPMO-centered strategy when grown on cellulosic substrates and more on canonical hemicellulases when grown on xylan. The LPMO-focused degradation approach is supported by co-regulation of other AA enzyme encoding genes that likely are expressed as LPMO co-factors. We also detected high GC and GC3 content as possible genomic characteristics contributing to the thermostability of the strain. The present study provides the basis for further biochemical characterization of the lignocellulose-degrading machinery in *T. terrestris* and filamentous fungi in general. The apparent complementary or redundant nature of certain CAZymes identified in this study needs to be investigated further with enzymological techniques, whereas a more detailed physiological understanding can be achieved with additional transcriptome and proteome studies.

## Methods

### Isolation and maintenance of fungi

Samples containing decaying plant residues (compost, grasses, rice straw, mushroom ground, wood, and soil) were collected from different provinces in Northern Vietnam during 2012–2016. Fungal strains were isolated as described by Thanh et al. [17] by incubation at 50 °C and under acidic conditions (pH 2.0) on medium containing untreated rice straw as the sole carbon source. After 7–10 days of incubation, fungal colonies were transferred to potato dextrose agar (PDA) plates and purified by hyphal tip culture at 50 °C. The isolates were maintained in PDA slants in a refrigerator at 2–8 °C.

### Growth on plates

Fungal strains were streaked out on solid base medium composed of 4 g L^−1^ KH_2_PO_4_, 13.6 g L^−1^ (NH_4_)_2_SO_4_, 0.8 g L^−1^ CaCl_2_‧H_2_O, 0.6 g L^−1^ MgSO_4_‧7H_2_O, 0.1 g L^−1^ peptone; 0.1 g L^−1^ yeast extract, 1000× trace element solution (10 mg L^−1^ FeSO_4_‧7H_2_O, 3.2 mg L^−1^ MnSO_4_‧H_2_O, 2.8 mg L^−1^ ZnSO_4_‧7H_2_O, 4 mg L^−1^ CoCl_2_‧6H_2_O, 3.5 mg L^−1^ CuSO_4_‧5H_2_O, pH 5.6), 1% (w/v) agar, and 2% (w/v) of one of the following carbon sources: Avicel, beechwood xylan, starch, guar gum, CMC, citrus pectin, cellobiose, d-glucose, d-xylose, locust bean gum, and inulin from Dahlia tubers or bark powder. Controls contained no carbon source. Plates were incubated at 30 °C or 50 °C for 1–7 days. Cellobiose was supplied by Megazyme. Bark powder was supplied by the Department of Chemistry and Chemical Engineering (Chalmers University of Technology, Gothenburg, Sweden) and contained 10% dried pine and 90% dried spruce bark. All other chemicals were supplied by Merck. Fungal strains were received from the collection at the Centre for Industrial Microbiology (Food Industries Research Institute, Hanoi, Vietnam).

### DNA and RNA extraction

To extract genomic DNA, strain LPH172 was grown on a PDA plate for 5 days at 50 °C, the mycelium was divided into six equal parts, and each part was used as inoculum in 100 mL liquid base medium containing 2% glucose. Cultures were incubated in 500-mL baffled Erlenmeyer flasks at 50 °C and 150 rpm for 48 h. The mycelium was harvested by filtering through sterile Miracloth (Merck Millipore) and rinsing extensively with liquid base medium without glucose. After pressing out excessive moisture by hand, the mycelium was snap-frozen in liquid nitrogen and ground to a fine powder in a TissueLyser (Qiagen) at 30-s, 30-Hz intervals with pre-cooled tungsten steel balls. CTAB buffer (2% CTAB, 100 mM Tris–HCl, pH 8.0, 20 mM EDTA, 1.4 M NaCl) was immediately added at 10 mL/g_mycelium_, briefly vortexed and the suspension incubated at 57 °C for 1 h. DNA was purified three times by phenol–chloroform extraction until no interphase was visible, followed by 2-propanol precipitation [75]. The resulting pellet was resuspended in 1 mL TE buffer (10 mM Tris–HCl, pH 8.0, 1 mM EDTA) and incubated with 200 μg mL^−1^ RNase A (Thermo Fisher Scientific) at 60 °C for 2 h to remove residual RNA. After an additional round of phenol–chloroform extraction, the pellet was resuspended in 150 μL TE buffer and DNA was further purified with the DNeasy Plant Mini Kit (Qiagen) according to the manufacturer’s instructions. Quality of the purified DNA was verified by agarose gel electrophoresis, Nanodrop (Thermo Fisher Scientific), and Qubit Fluorometer (Thermo Fisher Scientific) before genome sequencing.

For RNA extraction, a 100-mL pre-culture on glucose was prepared as described above for DNA extraction. After harvesting and washing the mycelium, this was divided equally between 250-mL baffled Erlenmeyer flasks containing 50 mL basal liquid medium supplemented with 2% Avicel, beechwood xylan, rice straw, corn cob xylan or glucose. After 5 days of cultivation at 50 °C and 150 rpm, the mycelium was harvested, frozen, and ground to a powder, as described for DNA extraction. RNA was extracted using TRIzol (Invitrogen) and chloroform, and further purified with the RNAeasy Plant RNA kit (Qiagen) with on-column DNAse digestion. Quality of the purified RNA was checked by agarose gel electrophoresis, Nanodrop (Thermo Fisher Scientific), Qubit Fluorometer (Thermo Fisher Scientific) and Bioanalyzer (Agilent Technologies). High quality RNA for transcriptome sequencing had and OD 260/280 of 1.8–2.0, an OD 260/230 of 2–0–2.2 and an RNA integrity number (RIN) of ≥ 8. Unless otherwise mentioned, all chemicals were supplied by Merck, except for corn cob xylan (Carbosynth) and rice straw powder (Center for Industrial Microbiology).

### Genome sequencing, assembly, and analysis

Genome sequencing and assembly was carried out by GATC Biotech (Constance, Germany). According to the company’s proprietary protocols, an 8–12-kb library was prepared by DNA fragmentation, size selection, end repair and adapter ligation, primer annealing, and polymerase annealing. Sequencing was performed on a PacBio RS II instrument (raw data output 400 Mb for a genome of ~ 37 Mb) with an average read length of > 6000 bp. De novo assembly of PacBio RS reads was achieved with proprietary GATC Biotech methods optimized for the sequencing technology, read length and type of raw data and included read filtering by length and quality, error correction of long PacBio reads through alignment of short reads (“reads of insert”), assembly of error corrected reads and assembly polishing. Completeness of the genome was assessed with BUSCO (v3.0.2b) against the fungi_odb9 gene dataset (http://buscodev.ezlab.org/datasets/fungiodb9.tar.gz). To analyze GC and GC3 content, seqinr, Biostrings, and sscu R packages were used [76–78].

### Transcriptome sequencing, assembly, and analysis

Transcriptome sequencing was performed by GATC Biotech (Constance, Germany) with the Inview Transcriptome Explore package. Briefly, a randomly primed cDNA library was prepared by purifying poly-A-containing mRNAs, fragmenting, adapter ligation, and PCR amplification. Illumina sequencing with single reads (50 bp) generated 24 million reads per sample that could be mapped to the reference. Quality checks were performed, and all samples achieved a percentage of clean reads > 95%. Assembly and annotation were done by National Bioinformatics Infrastructure Sweden (NBIS). Guided assembly was done with Tophat2 (v2.0.9) and Stringtie (v1.2.2), whereas repeat masking employed the RepeatModeler package (v1.0.8). *Ab initio* training for annotation was done with GeneMark-ET (v4.3), Augustus, and snap. Gene builds were computed using the MAKER package (v3.01.1), which employed the following software: exonerate (v2.4), Blast+ (v2.2.28), RepeatMasker (v4.0.3), Bioperl (v1.6.922), Augustus (v2.7), tRNAscan (v1.3.1), snap, and GeneMark-ET (v4.3). Functional annotation of genes and transcripts was performed using the translated CDS features of each coding transcript. For each predicted protein sequence, a BLASTp search was performed on the UniProt/Swiss-Prot reference dataset with default parameters (e-value cut-off = 1, similarity cut-off = 30%) to retrieve gene name and protein function. Secreted proteins were predicted using the SignalP 4.0 Server. Genes containing CAZy domains were identified using dbCAN2 (accessed October 2019). Reads were assigned and counted to the genome annotation using featureCounts of the Rsubread package (v2.2.6) in R and converted to TPMs. Differential gene expression was analyzed using edgeR (v3.30.3) [59] in R with TMM-normalization and removal of reads with less than 1 read per million in all samples.

## Supplementary Information

**Additional file 1:** Homologous sequences of transcription-elongation factor-alpha and beta-tubulin genes used for identifying the strain as *T. terrestris* LPH172 and phylogenetic analysis for the strain identification.

**Additional file 2:** Growth of *T. terrestris* LPH172 and other biomass-degrading filamentous fungi on different carbon sources.

**Additional file 3:** The complete list of putative CAZy domains detected in *T. terrestris* LPH172 and other filamentous fungi.

**Additional file 4:** Putative homologues of known transcription factors directly regulating (hemi)cellulose utilization in the genome of *T. terrestris* LPH172.

**Additional file 5:** The complete list of all expressed genes on the four tested substrates.

**Additional file 6:** The complete list of all upregulated putative CAZyme encoding genes on the four tested substrates.

## Data Availability

All data generated or analyzed during this study are included within the article and as Additional files 1, 2, 3, 4, 5 and 6. The genome assembly has been deposited at DDBJ/EMBL/GenBank under the assembly Accession No. GCA_900343105.1. Transcriptomic data are deposited at the European Nucleotide Archive (ENA) under the Accession No. PRJEB25201 (ERP107096). The strain is deposited in the Food Industries Research Institute (Hanoi, Vietnam) culture collection.
