# Supplementary material for: Genomic and transcriptomic analysis of the thermophilic lignocellulose-degrading fungus Thielavia terrestris LPH172
Source: Biotechnol Biofuels. 2021 Jun 3;14:131. doi: 10.1186/s13068-021-01975-1 (PMC8176577; doi:10.1186/s13068-021-01975-1)
Supplement: Supplementary file 1 — Additional file 1: Homologous sequences of transcription-elongation factor-alpha and beta-tubulin genes used for identifying the strain as T. terrestris LPH172 and phylogenetic analysis for the strain identification. [file 13068_2021_1975_MOESM1_ESM.docx]

**Additional file 1.**

Homologous sequences of transcription-elongation factor-alpha and beta-tubulin genes used for identifying the strain as *T. terrestris* LPH172.

**Method**

First, homologues of these genes from ascomycetous filamentous fungi were looked up in GenBank (KM655459.1 and M38265.1), then blasted with the *T. terrestris* NRRL 8126 coding sequences to find the matching homologues in *T. terrestris (*Thite2|2111611 and Thite2|2149383). Lastly, the homologues from NRRL 8126 were blasted with the *T. terrestris* LPH172, giving the results described in the article.

The TEF1 homologue in *T. terrestris* LPH172:

>THTET00000002213 gene=THTEG00000002213 name=tef seq_id=unitig_0|quiver type=cds

ATGGGCAAGGAAGACAAGACCCACATCAACGTGGTCGTCATCGGCCACGTCGACTCTGGC

AAGTCCACGACGACTGGTCACTTGATCTACAAGTGCGGTGGTATCGACTCCCGTACCATC

GAGAAGTTCGAGAAGGAAGCTGCCCAGCTCGGCAAGGGCTCCTTCAAGTATGCGTGGGTT

CTCGACAAGCTCAAGGCTGAGCGTGAGCGTGGTATCACCATCGATATCGCCCTCTGGAAG

TTCGAGACCCCCAAGTACATGGTCACTGTCATCGATGCCCCCGGCCATCGTGACTTCATC

AAGAACATGATCACTGGTACTTCCCAGGCCGACTGCGCTATTCTCATCATTGCCGCCGGT

ACTGGTGAGTTCGAGGCTGGTATCTCCAAGGATGGCCAGACTCGTGAGCACGCTCTGCTT

GCCTACACCCTGGGTGTCAAGCAGCTCATCGTCGCCATCAACAAGATGGACACGACCAAC

TGGTCCGAGGCTCGGTTCAACGAGATCATCAAGGAGACCTCGAACTTCATCAAGAAGGTC

GGCTACAACCCCAAGTCGGTCGCCTTCGTCCCCATCTCCGGCTTCCACGGCGACAACATG

CTGGAGCCCTCCGCCAACGCTCCCTGGTACAAGGGCTGGGAGAAGGAGGTCAAGAACGGC

AAGGTCACCGGCAAGACCCTCCTTGAGGCCATTGACGCCATCGAGCCCCCCAAGCGCCCC

ACCGACAAGCCCCTCCGCCTTCCTCTGCAGGATGTGTACAAGATCGGCGGTATCGGCACT

GTCCCTGTCGGCCGTATTGAGACCGGTATCCTGAAGCCCGGCATGGTTGTTACCTTCGCC

CCTTCGAACGTCACCACTGAAGTCAAGTCCGTCGAGATGCACCACGAGTCGCTCGCTGAG

GGTGTTCCGGGCGACAACGTCGGCTTCAACGTGAAGAACGTTTCCGTCAAGGAAATCCGC

CGCGGCAACGTTGCCGGCGACTCCAAGAACGACCCTCCTGCTGGCGCCGCTTCCTTCGAG

GCCCAGGTCATCATTCTCAACCACCCCGGTCAGGTCGGCGCTGGCTATGCCCCCGTCCTC

GACTGCCACACCGCGCACATTGCCTGCAAGTTCGCTGAGCTCCTCCAGAAGATCGACCGC

CGCACTGGTAAGGCCGTCGAGGACAACCCCAAGTTCATCAAGTCTGGCGATGCCGCCATC

GTCAAGATGATTCCCTCGAAGCCCATGTGCGTGGAGGCTTTCACCGAGTACCCTCCCCTT

GGTCGTTTCGCCGTCCGCGACATGCGTCAAACCGTCGCCGTCGGTGTCATCAAGAAGGTT

GAGAAGGCTGCTGCCGGTGCCGGCAAGGTCACCAAGTCCGCTGTCAAGGCCTCCAAGAAA

TAA

The beta-tubulin homologue in *T. terrestris* LPH172:

>THTET00000009462 gene=THTEG00000009462 name=tub-2 seq_id=unitig_90|quiver type=cds

ATGCGTGAGATCGTTCACCTCCAGACCGGCCAATGCGGTAACCAAATTGGTGCCGCTTTT

TGGCAGACCATCTCGGGCGAGCACGGCCTTGACAGCAATGGCGTGTACAATGGCACCTCC

GAGCTCCAGCTCGAGCGCATGAACGTCTACTTCAACGAGGCCTCCGGCAACAAGTATGTC

CCTCGTGCCGTCCTCGTCGACCTGGAGCCCGGCACCATGGATGCCGTCCGCGCTGGACCC

TTCGGCCAGCTCTTCCGCCCTGACAACTTCGTCTTCGGCCAGTCCGGTGCTGGCAACAAC

TGGGCCAAGGGTCACTACACCGAGGGTGCCGAGCTCGTGGACCAGGTCCTCGATGTCGTC

CGTCGCGAGGCCGAGGGCTGCGACTGCCTCCAGGGCTTCCAGATCACCCACTCGCTCGGT

GGTGGTACCGGTGCCGGTATGGGTACCCTCCTGATCTCCAAGATCCGCGAGGAGTTCCCC

GACCGCATGATGGCCACCTTCTCGGTCGTGCCCTCGCCCAAGGTCTCCGACACGGTCGTC

GAGCCTTACAACGCCACTCTCTCGGTGCACCAGCTGGTCGAAAACTCGGACGAGACCTTC

TGCATTGACAACGAGGCCCTCTACGACATCTGCATGCGCACCCTGAAGCTGTCGAACCCC

TCGTACGGTGACCTCAACTACCTGGTCTCGGCCGTCATGTCGGGCGTCACCGTCTCGCTG

CGCTTCCCCGGCCAGCTCAACTCGGACCTTCGCAAGCTGGCCGTCAACATGGTTCCTTTC

CCGCGTCTCCACTTCTTCATGGTTGGCTTCGCGCCTCTGACCAGCCGTGGCGCCCACTCG

TTCCGCGCCGTCTCCGTTCCTGAGTTGACCCAGCAGATGTTCGACCCCAAGAACATGATG

GCTGCGTCCGACTTCCGCAATGGTCGCTACCTCACCTGCTCTGCCATCTTCCGTGGCAAG

GTCTCCATGAAGGAGGTTGAGGACCAGATGCGCAACGTCCAGAACAAGAACTCGTCCTAC

TTTGTCGAGTGGATCCCGAACAACGTCCAAACGGCGCTGTGCTCGATCCCGCCGCGCGGT

CTCAAGATGTCGTCGACCTTCGTCGGCAACTCGACGGCCATCCAGGAGCTGTTCAAGCGT

GTTGGCGAGCAGTTCACGGCCATGTTCCGCCGCAAGGCCTTCTTGCACTGGTACACTGGC

GAAGGCATGGACGAGATGGAGTTCACCGAGGCCGAGTCCAACATGAACGATCTCGTCTCG

GAGTACCAGCAGTACCAGGATGCTGGCGTCGACGAGGACGAGGAGGGTTATGAGGAGGAG

GTTCCTGTCGAGCAGGAGGAGTAA

**Phylogenetic analysis**


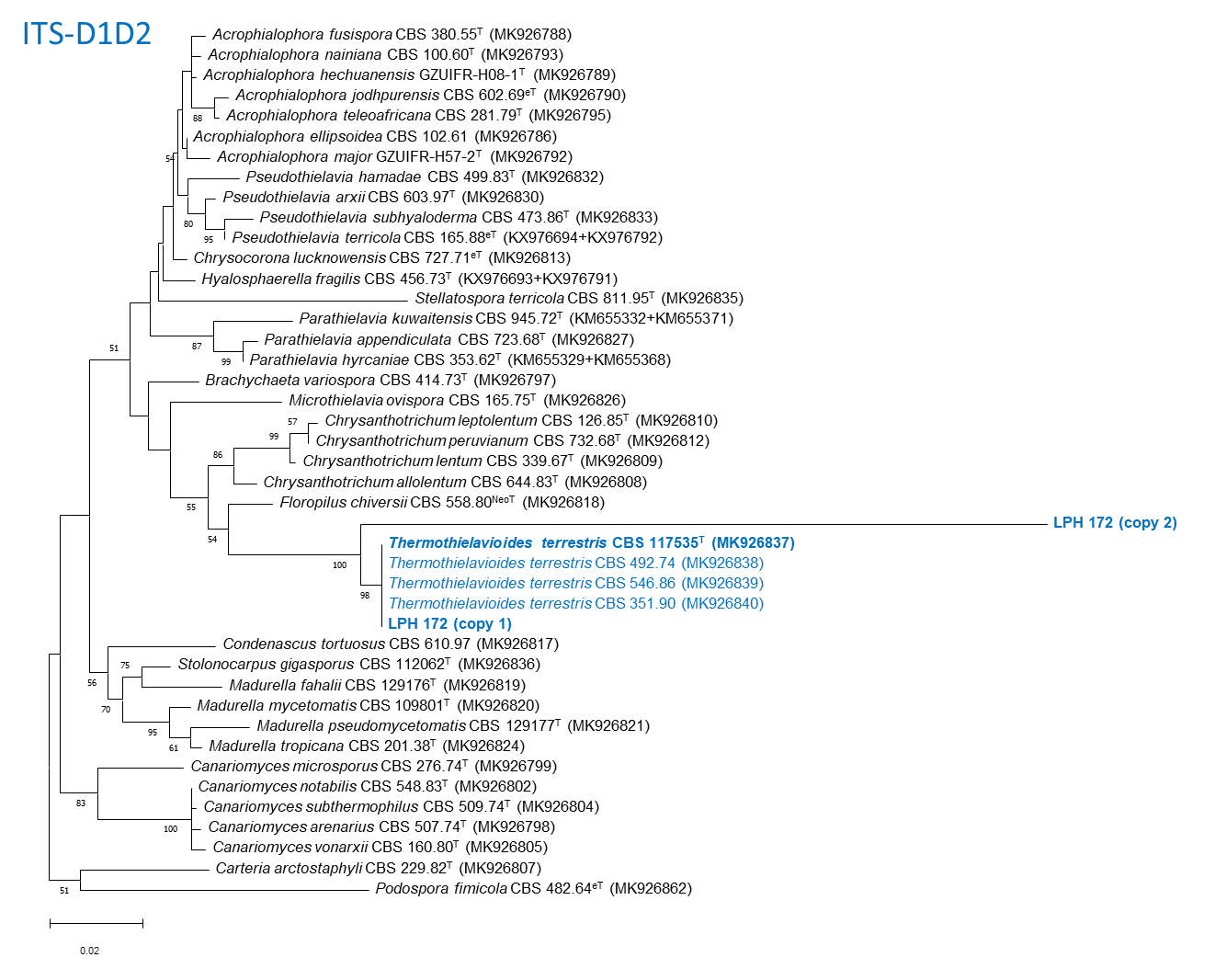


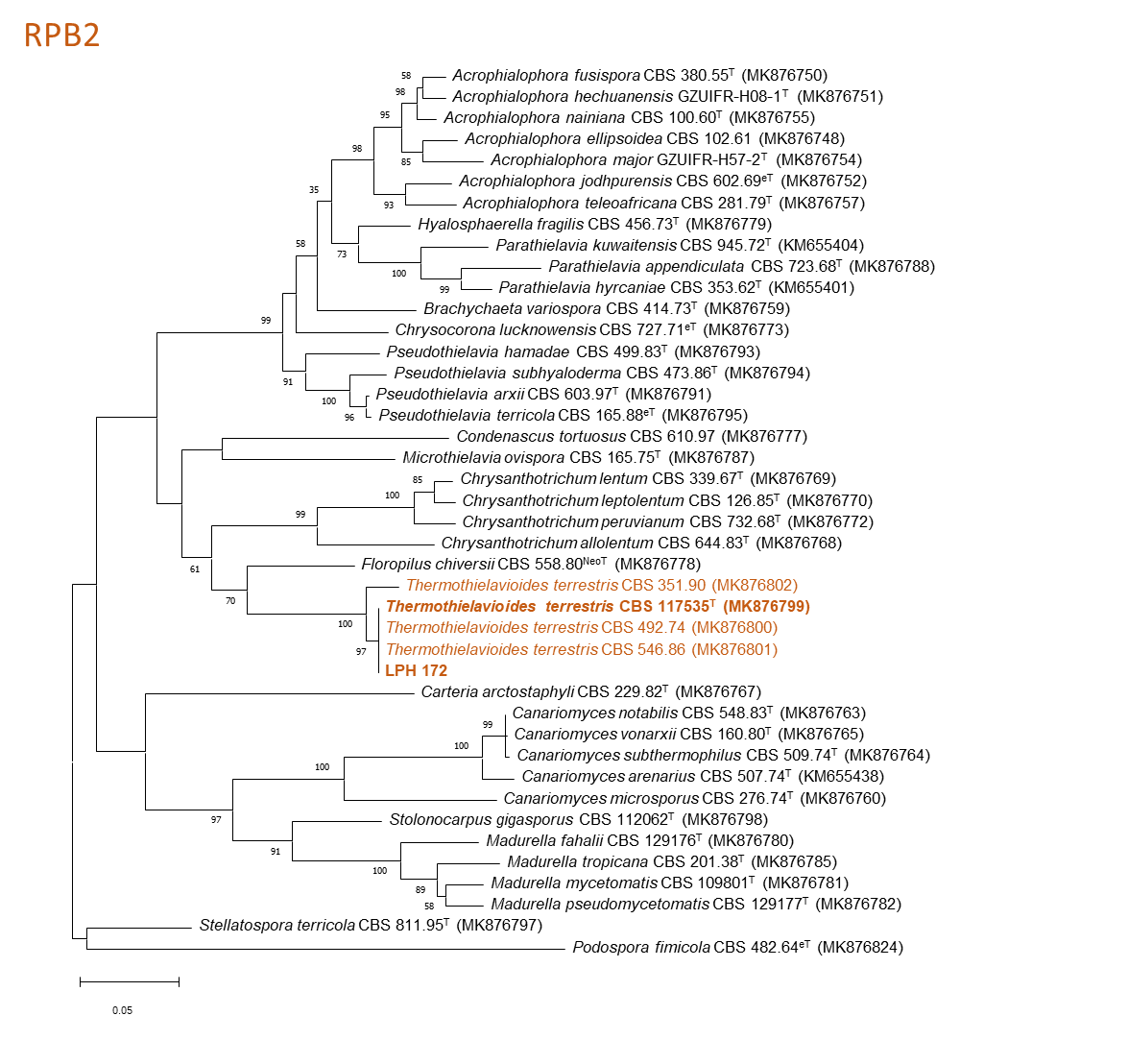


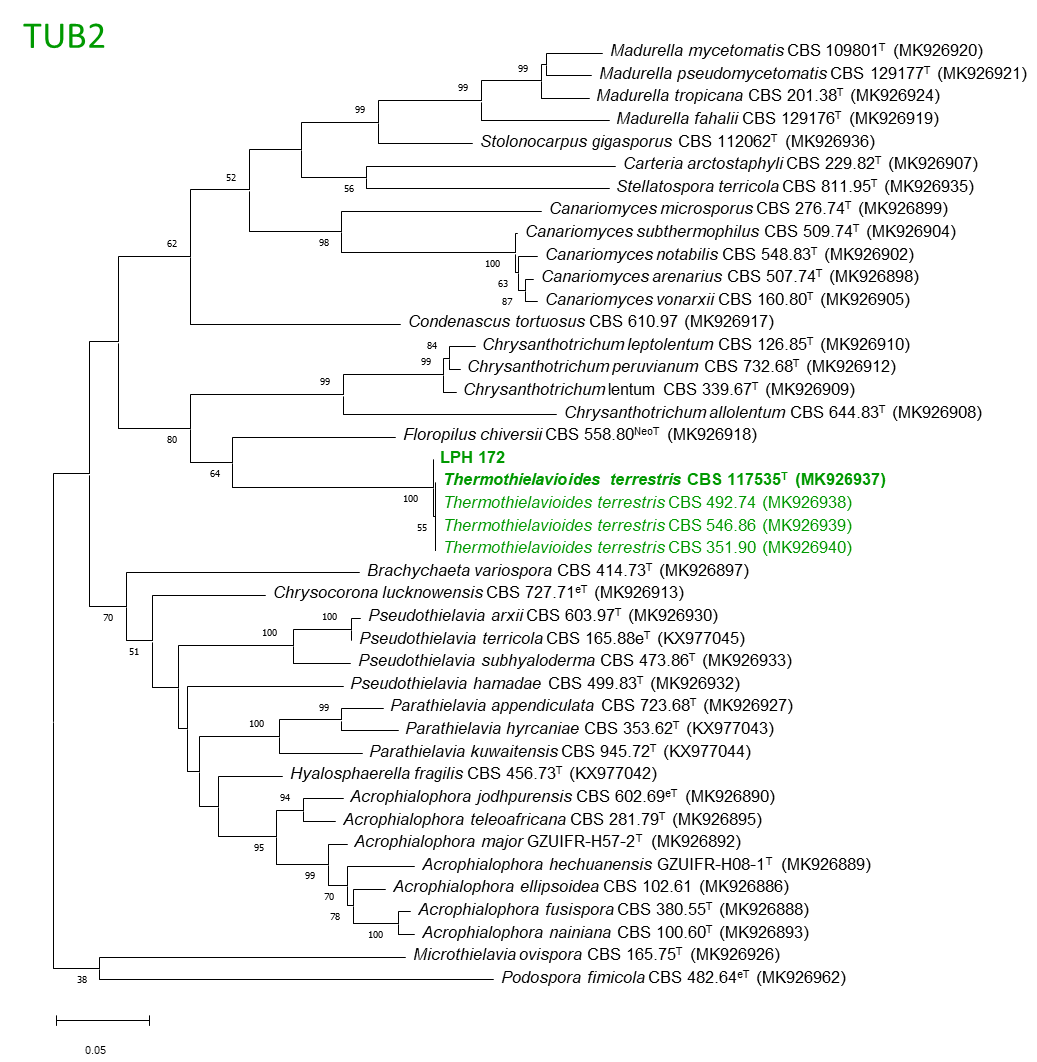


***Fig S1.*** Maximum Likelihood trees depicting the relationship between LPH 172 and related taxa of Chaetomiaceae. The trees were constructed based on the ITS-D1D2 (internal transcribed spacer and the D1D2 domains of the 28S nrDNA), RPB2 (a part of the DNA-directed RNA polymerase II second largest subunit gene), and TUB2 (the β-tubulin gene) sequences using Maximum Likelihood method and Tamura-Nei model [1]. Bootstrap values of >50 %, obtained from 1000 replications, are shown. All positions with less than 95% site coverage were eliminated. GenBank accession numbers of the sequences are given in parentheses. Evolutionary analyses were conducted in MEGA X [2].

*Note: LPH 172 sequences were extracted from the genome of the strain. There are two types of ITS-D1D2 sequences detected (“copy 1” and “copy 2”). Transcriptome analysis indicated that “copy 2” was most likely a pseudogene as it was not transcribed (while “copy 1” did). ITS-D1D2 (copy 1) and RPB2 sequences of LPH 172 were identical with the ones of CBS 117535T, the type strain of Thermothielavioides terrestris. There was single difference in the TUB2 sequence between two strains. Thus, LPH 172 could be reliably placed under Thermothielavioides terrestris.*

1. Tamura K. and Nei M. (**1993**). Estimation of the number of nucleotide substitutions in the control region of mitochondrial DNA in humans and chimpanzees. *Molecular Biology and Evolution* **10**:512-526.

2. Kumar S., Stecher G., Li M., Knyaz C., and Tamura K. (**2018**). MEGA X: Molecular Evolutionary Genetics Analysis across computing platforms. *Molecular Biology and Evolution* **35**:1547-1549.
