# Supplementary material for: Genomic and transcriptomic analysis of the thermophilic lignocellulose-degrading fungus Thielavia terrestris LPH172
Source: Biotechnol Biofuels. 2021 Jun 3;14:131. doi: 10.1186/s13068-021-01975-1 (PMC8176577; doi:10.1186/s13068-021-01975-1)

**Additional file 2.** **Growth of *T. terrestris* LPH172 and other biomass-degrading filamentous fungi on different carbon sources.** Twelve different carbohydrate substrates at 2% (w/v) were used as sole carbon sources for growth on agar plates: monosaccharides (glucose, xylose), disaccharides (cellobiose), and polysaccharides (starch, Avicel, carboxymethyl cellulose - CMC, beechwood xylan, pectin, locust bean gum, guar gum, inulin and bark powder). No carbon source was added in the control. The plates were incubated at 30°C (*S. commune*, *A. oryzae*) or 50°C (*M. thermophila*, *M. cinnamomea, T. terrestris*) for 2-7 days.


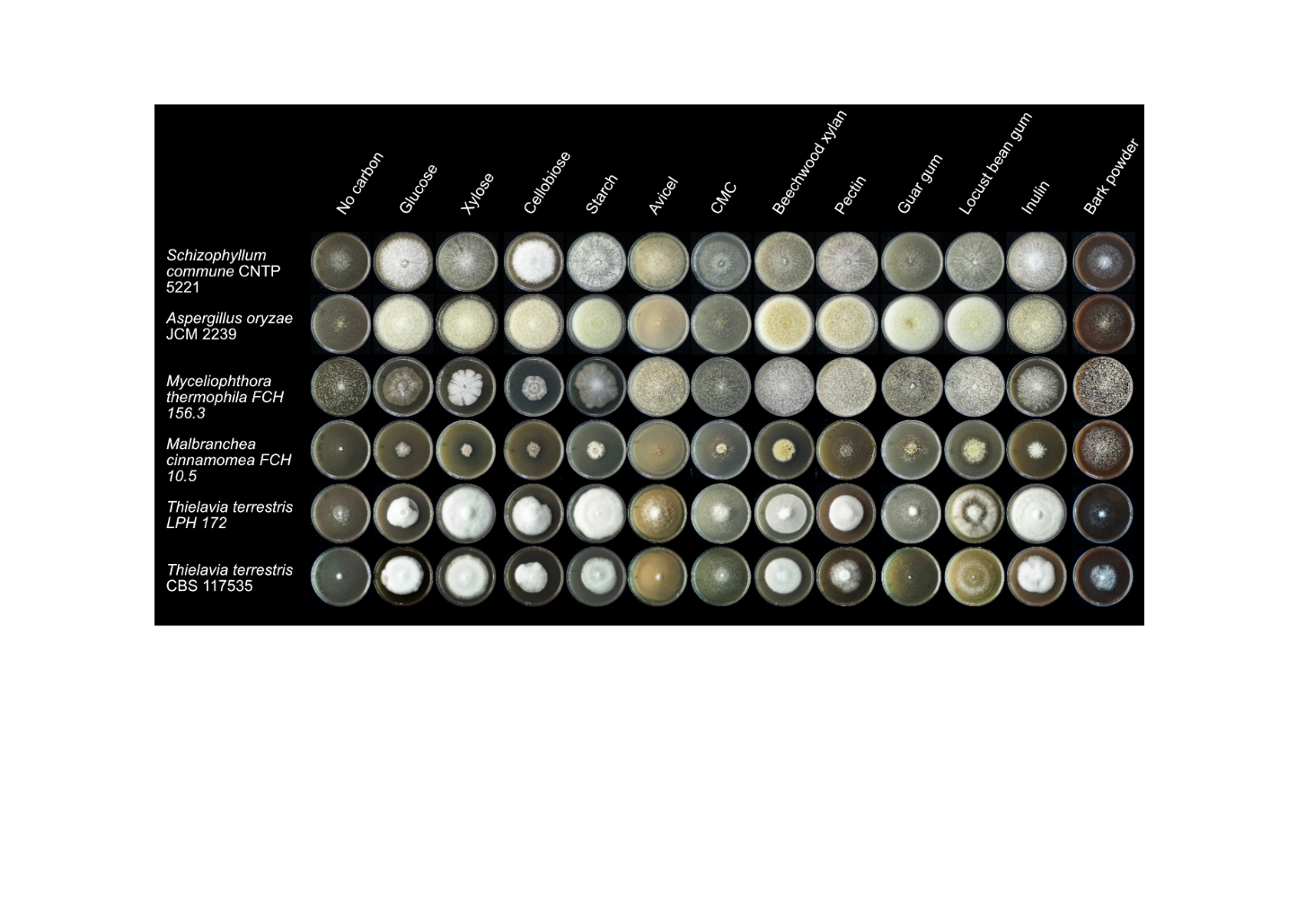

Supplement: Supplementary file 2 — Additional file 2: Growth of T. terrestris LPH172 and other biomass-degrading filamentous fungi on different carbon sources. [file 13068_2021_1975_MOESM2_ESM.docx]
